# Dynamic interplay between available resources and implementation climate across phases of implementation: a qualitative study of a VA national population health tool

**DOI:** 10.1186/s43058-023-00460-0

**Published:** 2023-06-29

**Authors:** Ying-Jen Lin, Allison Ranusch, F. Jacob Seagull, Jeremy B. Sussman, Geoffrey D. Barnes

**Affiliations:** 1 Center for Bioethics and Social Sciences in Medicine, University of Michigan, Ann Arbor, MI USA; 2 Center for Clinical Management Research, Veterans Affairs Ann Arbor Healthcare System, Ann Arbor, MI USA; 3 Institute for Healthcare Policy and Innovation, University of Michigan, Ann Arbor, MI USA; 4 Division of General Medicine, Department of Internal Medicine, University of Michigan, Ann Arbor, MI USA; 5 Frankel Cardiovascular Center, Department of Internal Medicine, University of Michigan, 2800 Plymouth Rd, B14 G214, Ann Arbor, MI 48109-2800 USA

**Keywords:** Available resources, Implementation climate, Technology-based intervention

## Abstract

**Background:**

Available resources within an organization can determine the implementation success of an intervention. However, few studies have investigated how the required resources change over the phases of implementation. Using stakeholder interviews, we examined the changes in and interactions between available resources and implementation climate in the implementation and sustainment phases of a national implementation effort for a population health tool.

**Methods:**

We conducted a secondary analysis of the interviews with 20 anticoagulation professionals at 17 clinical sites in the Veterans Health Administration health system about their experiences with a population health dashboard for anticoagulant management. Interview transcripts were coded using constructs from the Consolidated Framework for Implementation Research (CFIR) and according to the phase of implementation (pre-implementation, implementation, and sustainment) as defined by the VA Quality Enhancement Research Initiative (QUERI) Roadmap. We analyzed the factors that may determine successful implementation by examining the co-occurrence patterns between *available resources* and *implementation climate* across different implementation phases. To illustrate the variations in these determinants across phases, we aggregated and scored coded statements using a previously published CFIR scoring system (− 2 to + 2). Key relationships between *available resources* and *implementation climate* were identified and summarized using thematic analysis.

**Results:**

The resources necessary to support the successful implementation of an intervention are not static; both the quantity and types of resources shift based on the phases of the intervention. Furthermore, increased resource availability does not guarantee the sustainment of intervention success. Users need different types of support beyond the technical aspects of an intervention, and this support varies over time. Specifically, available resources in the form of technological support and social/emotional support help users establish trust in a new technological-based intervention during the implementation phase. Resources that foster and maintain collaboration between users and other stakeholders help them stay motivated during sustainment.

**Conclusions:**

Our findings highlight the dynamic nature of *available resources* and their impacts on the implementation climate across different phases of implementation. A better understanding of the dynamics of *available resources* over time from the users’ perspectives will allow the adaptation of resources to better meet the needs of the intervention stakeholders.

**Supplementary Information:**

The online version contains supplementary material available at 10.1186/s43058-023-00460-0.

Contributions to the literature

- The quantity and types of resources required for supporting users of a technology-based intervention shift throughout the phases of the implementation process.
- Increased resource availability does not guarantee the successful sustainment of an intervention.
- In the implementation phase, users of the technology-based intervention need resources in the form of technological support and social/emotional support that help them establish trust in the intervention.
- In the sustainment phase, key resources are those that foster and maintain collaboration between users and other implementation partners. These resources help users stay motivated to use the intervention.

## Background

The availability of organizational resources, including funds, training, staffing, technological support, and time, dedicated to the implementation and ongoing operations of interventions has received long-standing attention in implementation science [1–5]. The level of available resources is often conceptualized as an independent variable that can influence the success or failure of the implementation outcomes (the dependent variable) in determinant frameworks, such as the Promoting Action on Research Implementation in Health Services (PARIHS) [6, 7], the conceptual model [4], and the Consolidated Framework for Implementation Research (CFIR) [8].

### Available resources

While the existing literature predominantly views available resources as one factor that determines the success or failure of an intervention, most studies focus on the impact of available resources on implementation as a whole or focus primarily on the early stage of implementation [9–12]. Research that used determinant frameworks may not explicitly take a process perspective of implementation and fail to recognize the changing implementation needs over time [13]. In reality, the amount and type of resources needed to plan and execute implementation strategies and adapt the intervention to the clinical context are all expected to change over the course of an intervention [14]. This is particularly true for technology-based interventions in healthcare settings due to the dramatic alterations from technical to social problems during technological adoption. As the implementation process unfolds over time, users of interventions may encounter different kinds of implementation challenges that require different types of support and resources. These qualitative shifts within a resource type have not been adequately studied.

### Implementation climate

Understanding the users’ perspectives is essential to the implementation success as the users hold first-hand knowledge of which available resources or facilitating factors they need to better integrate the intervention into their daily work environments [15, 16]. To assess the effectiveness of an intervention from the users’ perspectives, researchers in implementation science have turned their attention to defining and measuring implementation climate [17–20]. Implementation climate refers to the extent to which intended users perceive the use of the intervention as expected, supported, and rewarded within their organizations [21]. Over 25 years, implementation climate has been shown to be a vital component of implementation success [17, 22–25]. While there are a few studies investigating whether and how implementation climate changes over time, they have focused primarily on the effect of leadership on implementation climate [24, 25]. Just as understanding the dynamic changes in resource use requires more empirical data describing resource demands over time, research on implementation climate may benefit from more data regarding how the effect of different resource types on implementation climate changes over the course of the intervention.

### DOAC Population Management Dashboard

This study examines the dynamic relationships between available resources and implementation climate using the DOAC Population Management Dashboard as an example. The dashboard is an innovative technological intervention that leverages clinical data from electronic health records (EHR) to improve the safe prescribing and management of direct oral anticoagulants (DOACs). It updates the traditional care model to a population-based management approach, allowing oversight of all patients prescribed DOACs and intervening only when clinically necessary [26]. Studies show that it improves patient safety and pharmacy efficiency, reduces adverse events, and increases clinic access [27, 28]. The DOAC Dashboard is an effective technological intervention for evidence-based anticoagulation medication management that can be implemented nationwide in various clinical contexts [29].

In our previous qualitative research on the implementation process of the DOAC Dashboard, we identified five key determinants that affect implementation success: (1) clinician authority and autonomy; (2) clinician self-identity and job satisfaction; (3) documentation, communication, and administrative needs; (4) staffing and work schedule; and (5) integration with existing information systems [30]. Our previous study implicitly recognized the significance of available resources and implementation climate. However, we did not specifically investigate how these factors may change over time, especially during the transition from the implementation phase to the sustainment phase. This knowledge gap is critical because the dynamics of resource availability and implementation climate can significantly impact the long-term success and sustainability of technological interventions in healthcare settings.

As the implementation of technological interventions in healthcare settings continues to increase, it is crucial to gain a deeper understanding of the interplay between available resources and implementation climate. The effective deployment of available resources requires a comprehensive understanding of their demand, allocation, and the specific contextual factors that influence their utilization. Therefore, there is a need for empirical insights to inform policy and practice in this domain. To address this need and advance our understanding of the role of available resources and implementation climate, as well as their dynamic interplay, this study utilized a secondary analysis approach. We analyzed interviews from our previous research and applied CFIR and a process perspective to a nationwide implementation effort for the DOAC Population Management Dashboard. Specifically, this study aims to answer the following questions: (a) How do *available resources* and *implementation climate* shift across different phases of implementation? (b) How do the changes in *available resources* affect *implementation climate* in various stages of implementation?

## Methods

### Research setting and participants

We used interview data derived from our previous research on nationwide implementation efforts of the DOAC Population Management Dashboard in the United States Veterans Health Administration (VA) [30]. The VA health system offers healthcare services to more than 9 million enrolled veterans each year [31]. The VA’s anticoagulation clinic services employ pharmacists who are supported by the DOAC Population Management Dashboard to help identify and reduce the potentially unsafe use of DOACs [29]. The DOAC Population Management Dashboard had been available to most VA pharmacists for up to 3 years at the time of the interviews, which were conducted between November 2019 and November 2020. We used a comparative multiple-case study design with the goal of analyzing the patterns across different VA clinical sites in order to produce more generalizable knowledge about whether and how available resources and implementation climate change across implementation phases specifically for technological interventions in healthcare settings. This study was approved by the institutional review board of the Ann Arbor VA Healthcare System. Interview participants were identified by clinic managers and invited to participate via e-mail communication. The semi-structured interview guide was created using predefined constructs from the Consolidated Framework for Implementation Research (CFIR) [8] and the Technology Acceptance Model (TAM) [32] (Additional file 1). The interviews and data analysis were conducted by the research staff with training in qualitative research. The average length of interviews was 37 min (range 25–57 min). Each transcript was de-identified prior to coding and analysis.

### Qualitative data coding

We used the qualitative analysis software MAXQDA [33] to code and analyze the existing interview data. Data coding and analysis were guided by the VA Quality Enhancement Research Initiative (QUERI) Roadmap and the CFIR.

The interview transcripts were first coded by implementation phases adapted from the VA QUERI Roadmap, including the phases of pre-implementation, implementation, and sustainment [34]. We coded participants’ responses regarding their implementation efforts prior to the adoption of the DOAC Population Management Dashboard as the *pre-implementation* phase, including the process of identifying problems and potential solutions to optimize anticoagulation management, engaging stakeholders, and assessing organizational capacity prior to implementing the dashboard. Participants’ responses would be coded as the *implementation* phase if they discussed how the dashboard was first introduced to their clinics and the process of learning the new tool, activating the implementation teams, getting technical and adaptive support, and cleaning the backlog of notifications in the early phase of using the DOAC Population Management Dashboard. We coded participants’ statements as the *sustainment* phase if they were related to whether and how the dashboard has been embedded and become routine practice in their clinical settings as well as their ongoing evaluation and reflection regarding the impact of the dashboard on their anticoagulation practice.

When we coded interview data using the CFIR, we focused on two specific CFIR codes in the inner setting domain: *available resources* and *implementation climate*. In the initial stage of coding, we discovered that certain elements of available resources mentioned by participants were closely related to other CFIR constructs, such as *champions* and *leadership engagement*. Consequently, to capture this relationship, we expanded the definition of available resources to encompass the support provided by champions, leaders, and other stakeholders involved in the implementation process. When examining the CFIR code of *implementation climate*, we also included the six sub-constructs of implementation climate, including tension for change, compatibility, relative priority, organizational incentives and rewards, goals and feedback, and learning climate [8]. The expanded definition of available resources, along with the comprehensive exploration of the implementation climate and its six sub-constructs were applied when coding the transcripts and assigning ratings to the CFIR constructs (as described in the “Rating with CFIR constructs” section).

To achieve consistency during the coding process, three researchers (AR, FJS, and YL) independently coded the same transcripts and met to discuss the overlaps and discrepancies in coding after each transcript. After independent coding and discussion of five transcripts, coding was consistent across all coders. Upon achieving consensus, we divided the remaining transcripts among the team for individual coding. The team continued to meet and discuss any questions that came up during our individual coding process and used consensus to resolve any statements that were perceived as ambiguous or difficult to code clearly.

### Rating with CFIR constructs

Once coding was complete, we examined the intersection between the three implementation phases (pre-implementation, implementation, and sustainment) and the CFIR codes. Using MAXQDA, all coded segments were aggregated, sorted by the interviewee’s site and study ID, and grouped by phase. We reviewed the coded segments from the interview data and created a document containing all summary statements, plus supporting quotes for each interview.

The coded segments in the document were rated using the previously published CFIR rating system [11, 35]. Three researchers (AR, FJS, and YL) first rated the same cases to ensure the consistency of the rating. After rating each case independently, we discussed the ratings until a consensus was reached. After two cases, the ratings were consistent across raters. We then divided the remaining cases and rated them independently. During the rating process, the three researchers continued to meet to resolve any potential discrepancies.

We created a table to place two CFIR constructs (*available resources* and *implementation climate*) in the columns and three implementation phases (pre-implementation, implementation, and sustainment) in the rows for each site to facilitate the rating of segments where implementation phases and CFIR constructs overlapped. In accord with the CFIR scoring system [11, 35], interview segments with the two CFIR codes were rated with a 5-point bipolar scale (− 2 to + 2) to reflect the valence (positive or negative) and magnitude of changes in *available resources* and *implementation climate* over the course of implementation. Interviews with the majority of statements that were positive toward a construct’s influence on the implementation process were assigned a score of + 2 or + 1 (facilitator) depending on the strength or magnitude of the construct discussed by the interviewee. Similarly, interviews with negative statements were assigned a score of − 2 or − 1 (barrier). An equal mixture of positive and negative statements received a 0 score (neutral) for that CFIR construct.

When there were various dimensions of CFIR constructs, such as different types of *available resources* or sub-constructs of *implementation climate*, we rated each item individually but also took into account the relative importance of each item. A weighted average was generated and assigned to the two CFIR constructs to reflect the relative importance of some dimensions of *available resources* or *implementation climate* at each site. We excluded the data of CFIR constructs during the pre-implementation phase from this analysis because too many participants either were not involved in the pre-implementation phase or were not knowledgeable about the available resources prior to the implementation.

### Thematic analysis

After the rating process was complete, we used a graph to visualize the way that *available resources* and *implementation climate* change from implementation to sustainment phases for each site. We categorized sites based on the direction of change in the two CFIR constructs across phases. We also conducted a qualitative thematic analysis across three groups, as specified in the results, to identify the key resources that affected the implementation climate at different phases and examine how available resources and implementation climate interacted with each other during these phases.

## Results

### Interviewee characteristics

In this study, we focus on anticoagulation professionals’ experiences of using the DOAC Population Management Dashboard during both the implementation phase and sustainment phase at the VA clinical sites across the USA. We analyzed transcripts of 20 semi-structured phone interviews conducted with anticoagulation professionals at 17 VA clinical sites, which had reached the sustainment phase of implementation of the DOAC Population Management Dashboard. Interview participants included 13 pharmacists, one pharmacy technician, and six pharmacy clinic managers. Regarding staff involvement in the intervention, 18 out of 20 interviewees shared details about their duration of engagement with the intervention. The average length of their involvement, as reported at the time of the interview, was 2 years. Furthermore, 95% of the interviewees reported being the initial users of the DOAC Dashboard since its adoption by their clinics.

### Changes in resource availability and implementation climate over time

In our previous research interviews [30], we explored the barriers encountered during the implementation of the DOAC Dashboard. By analyzing participants’ responses, we were able to gain valuable insights into the resources or support they used to overcome these barriers. During the initial analysis of the transcripts, we found that certain available resources mentioned by the participants were closely connected to other CFIR constructs, such as *champions* and *leadership engagement*. For example, several participants mentioned that champions played an important role in offering training and other needed resources; time and staffing dedicated to the intervention were closely linked to formal leadership support. We therefore expanded our definition of available resources to incorporate the support from stakeholders, such as champions, leaders, and other implementation partners to fit the context of DOAC Dashboard implementation.

**Fig. 1:**
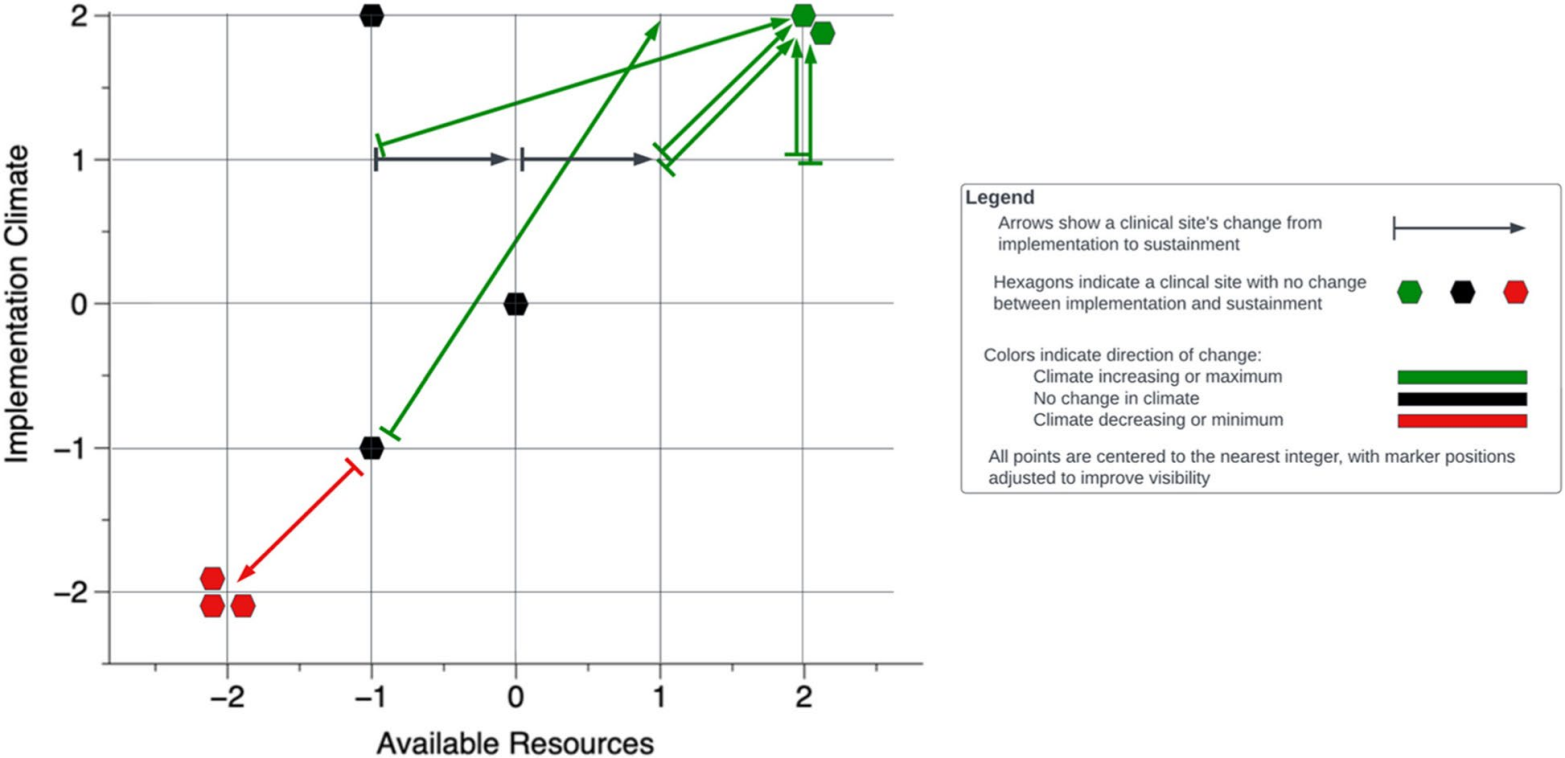
Changes in implementation climate and available resources from the implementation phase to the sustainment phase

To understand how *available resources* and *implementation climate* vary over time and across clinical sites, we graphed each site’s scores on these two parameters from implementation to sustainment phases (Fig. 1). This allows us to compare the “direction of movements” of the relative positions of the two CFIR constructs among various sites.

In the early phase of implementation, the sites in the study had a wide variety of available resources and implementation climate with scores ranging from − 2 to + 2 for both measures. Among the 17 sites, nine sites (green arrows and dots) had a more positive implementation climate in the sustainment phase. These sites generally had an increase in resource availability or maintained the same level of high resource availability (+ 2) over the trajectory of the implementation process. Four sites (black arrows and dots) had no change in implementation climate while their resource availability increased or maintained the same moderate levels (− 1 to 0). The sites with increased resources but no improvement in implementation climate (black arrows) are further examined in the following section (group 2). Four sites out of 17 had a decreased level of implementation climate (or remained the lowest (− 2)) on this dimension, and these sites all had the lowest level of resource availability in the sustainment phase.

### The interplay between available resources and implementation climate

Based on the groupings in Fig. 1, we conducted a thematic analysis to examine what types of available resources influenced the implementation climate in the implementation phase and the sustainment phase.

#### Group 1: Sites with positive changes in both available resources and implementation climate

We first focused on the sites with positive changes in both resource availability and implementation climate across phases (Table 1). Champions, time, staffing, and leadership support during the implementation phase were identified as key resources that facilitate the adoption of the intervention at the clinical sites of those interviewed.

**Table 1:** Examples of resource-climate interactions at group 1 clinical sites

Available resources	Implementation climate	Interplay	Phase	Illustrative quote
Champions (training, technological and social/emotional support)	Staff’s receptivity and trust in the new intervention	**Champion (↑) → trust (↑)** A champion (the intervention developer) has helped the intervention users understand the reasoning behind the design of the intervention, and develop trust in the new tool	Implementation	“After all this time of using it, I have to say I know what the rules are in and out, and I talked to [a developer of the intervention] a lot, so I feel like I understand his reasoning behind a lot of the codes, so I definitely, I trust it.” Site 1, Study ID 5
		**Support (↑) → receptivity (↑)** It is important to have a champion available for people to express their frustrations to and who can empathize with them and understand their pain points		“it’s really important to have one person I think as kind of the key person maybe or just get somebody onboard who can show the benefit of it, and then just keep discussing it and have open conversations… If people are really mad, just let them vent a little bit and how can we work through that…” Site 45, Study ID 173
	Learning climate	**Education resources (↑) → learning (↑)** The interviewee helped create training materials for co-workers who volunteered to learn the dashboard and use it when the interviewee was unavailable. Because of the training materials, the staff can “fumble through” the dashboard		“I think most of them used that PowerPoint that I put together when they have to cover, and then they just go through that, like, ‘Okay, first I do this, and then I do that,’ and yeah, just go about it that way…they can fumble through it using like my how-tos.” Site 30, Study ID 175
Trialability (the ability to experiment with the new tool)	Staff’s receptivity and trust in the new intervention	**Time (↑) → trust (↑)** The staff were initially uncomfortable with relying on the dashboard, so they created a hybrid system in which they ran the dashboard but also did periodic patient reviews. They realized the dashboard was catching issues before their reviews were scheduled, as well as issues they would not have been prompted to check at all. Through this hybrid process, they realized that the dashboard is a beneficial tool and they established trust in the new tool	Implementation	“… we had kind of a hybrid model for probably about six months after going to the dashboard and I think by that point the pharmacists who weren’t really fully on board with it, they realized that there were alerts being generated in between the time they had, you know planned to follow-up with the patient, and they noticed that there were things that clinically really needed to be addressed before that six month mark or that 12 month mark but there wouldn’t have been any other reason for them to get into that veteran’s chart prior to then to review for any issues. So, I think that proved to them that the dashboard really was picking up on very critical things that needed to be addressed in a timely manner that our previous system would’ve missed… So, probably I would say at least a year after we started, everybody was fully onboard just doing the dashboard.” Site 30, Study ID 175
Time, staffing, and supportive leadership	The extent to which the use of the intervention was supported within their organization	**Staffing (↑) → barriers (↓)** The initial time investment in cleaning up the backlog of alerts made the dashboard more usable for them in the sustainment phase. The staff were able to overcome the initial challenge by recruiting more helps, which also required the leader’s support	Implementation	“So that initial review for a lot of the flags was very difficult just because we had to go through so many of them. That was probably the most difficult part of implementation, and we actually recruited some help from our pharmacy residents, we recruited some help from our Primary Care pharmacists, from our Cardiology pharmacists. We had a lot of our pharmacists taking a look at this dashboard all at the same time just to get the initial review done to the point where… let’s say if you have 500 flags for you know critical drug interactions, you’re not going to be able to catch a meaningful flag that pops overnight. So, we really needed to get it down to the point where it would actually be meaningful to be looking at this on a day-to-day basis, which it is now at this point.” Site 44, Study ID 172
Time, staffing, and supportive leadership	Goals and feedback	**Feedback (↑) → resources (↑)** Staff did not want to admit that sometimes they were struggling to keep up with the dashboard. Eventually, they realized that the dashboard required more dedicated time to be successfully managed so they provided feedback to their leaders and hired new staff to resolve the issue	Sustainment	“…when we transitioned to the DOAC dashboard as it is now, and as it continued to evolve and become more comprehensive, it just flagged more patients and it just took more time… maybe that lag in us figuring out okay wait we need to spend more time on this. So, I mean to our management’s credit, I think that they really listened, and they tried to fix it.” “At first, people would kind of make excuses like, ‘Oh no, I can get it done, I just didn’t get it done this time because of this, this, this,’ and it was kind of like a self-protection thing, like, “I’ll get my job done, I’ll get it done’… I think as more people realized nobody is getting it done, then people felt more comfortable saying, ‘Yeah, it’s not me, it’s like the process, we need to have more time dedicated.’ So, I think there was that gap in there too, but then once people really started to be vocal about not being able to get it done, I would say probably 6 months to a year, you know by the time we hired that extra person and restructured the anti-coag clinic grid.” Site 13, Study ID 108
Consistent support from implementation partners	The extent to which the use of the intervention was supported within their organization	**Support** (**↓) → **c**limate (↓)** The use of the intervention has been supported within their organization most of the time, but the staff wished that more of their recommendations about the medications would be taken by physicians (implementation partners)	Sustainment	“I would like to see a greater change in you know, I wish that our interventions that we recommend, that more of those recommendations were taken.” Site 25, Study ID 112 “[Responding to what needs to happen to make the physicians more willing to take pharmacists’ recommendations] I think maybe more provider education. It would have to be probably a change in kind of I guess perspective. Maybe if the providers understood the training that we had as far as the anti-coag clinic pharmacists are supposed to be the expert in anti-coagulation but a lot of times we’ll get pushback and say, ‘Well Cardiology recommended this.’ Well okay, but Cardiology is recommending a dose that’s not recommended. So maybe education to the providers about the qualifications of the staff or just discussing with the staff that to make recommendations that are appropriate for the patient and not necessarily what was recommended by an outside provider or a cardiologist and just taking that as the gold standard.” Site 25, Study ID 112

##### Champions

Several participants from these sites mentioned that certain champions, such as intervention developers or experienced users, played an important role in providing support to help them trust and use the DOAC Population Management Dashboard. The participants stated that despite initial concerns about the reliability of the intervention, the support provided by champions during the implementation phase helped them develop trust and acceptance of this new tool. Notably, the champions’ support, according to the participants, not only included help resolving technical problems but also an in-depth explanation of the logic behind various actions on the dashboard; moreover, the participants found it valuable that the champions empathized with them and understood their pain points. Meanwhile, participants reported that no formal training was provided when they first started to use the dashboard at their VA clinical sites. As a result, some of the participants learned and navigated the intervention by themselves during the implementation phase. They then helped create training materials or step-by-step guidelines for other colleagues who implemented the intervention.

##### Time, staffing, and leadership

Whether there were sufficient time and staff for implementing the DOAC Population Management Dashboard greatly influenced the implementation climate during the implementation phase. At some sites, for example, the pharmacists continued using their previous system while implementing the new tool to verify that the new tool was providing accurate alerts and catching all potential errors for their patients. In such cases in which participants and their colleagues were able to experiment with the new tool, the resistance to the intervention tended to diminish over time as the users could verify the accuracy of the dashboard. Participants also highlighted the need for time and staff when faced with the challenge of processing an initial backlog of patient alerts in order to establish the ideal practice setting in which the alerts could be reviewed and managed daily by anticoagulation professionals. According to some participants, it took a significant amount of time or recruitment of extra help to review and process the initial backlog of alerts. They reported that to accomplish that, their leaders at the clinical sites often played an essential role in the deployment of necessary resources.

In the sustainment phase, time and staff remained key resources that affected the implementation climate. While some clinical sites were able to quickly activate existing staff or recruit extra help to learn and implement the intervention, many sites found it challenging to integrate the dashboard into their workflow during the implementation phase. According to the participants, as they already worked on various medication management tasks and patient care services, such as walk-in patient consultations, the dashboard often took a lower priority at their clinical sites. The lack of dedicated staff and time gradually surfaced as a major barrier to the sustainment of intervention over time. To overcome this barrier, these clinical sites ended up hiring staff that had dedicated time for dashboard management in the sustainment phase.

##### Support from partners

The support from implementation partners was also a critical factor that influenced the implementation climate during the sustainment phase. Since the DOAC Population Management Dashboard is designed for the use of anticoagulation professionals (pharmacists or nurses) to monitor safe outpatient anticoagulant prescribing by physicians and other clinicians, prescribers’ attitudes toward the recommendations made by the anticoagulation professionals greatly affected the implementation climate at the clinical site. According to one interviewee, despite feeling supported by the clinical site most of the time, the physicians’ preference for individual clinical judgment over the guidelines on the management of DOACs still caused frustration.

#### Group 2: Sites with increased resources but no improvement in implementation climate

We identified instances where available resources increased as the intervention moved from the implementation phase to the sustainment phase; however, the implementation climate did not improve (Table 2).

**Table 2:** Examples of resource-climate interactions at group 2 clinical sites

Available resources	Implementation climate	Interplay	Phase	Illustrative quote
Staffing (more staff were involved, but no dedicated staff/time)	Compatibility and relative priority (integration of the dashboard into existing workflows)	**Staffing (↑) → climate (≈)** More staff were involved in the intervention from implementation to sustainment phases due to their facility policy; however, the staff could not always get to the intervention because they had to juggle too many other dashboards. The interviewee suggested that having a dedicated group and dedicated time for intervention might help resolve the problem	Sustainment	“it was in the fall of 2016 when we started to use it and it was really just me using it as the anticoagulation program manager… as we have more and more patients switched to a DOAC, it needs to be a multiple person you know job, not just me because I wasn’t keeping up with it as I should have and since 2016, there’s been several more columns and types of flags added too, which is helpful in identifying those patients that may require a re-evaluation of dose or the critical drug interaction… something like that.” “… we may not always get to the DOAC dashboard every week because we have so many other dashboards to be using and addressing for population management. So, I think myself in our group, we would prefer to have a separate anticoagulation pharmacist group and a separate PACT pharmacist group so that we could manage these dashboards a little bit better and more effectively and efficiently.” Site 28, Study ID 118
Time (staff’s downtime)	Compatibility and relative priority (integration of the dashboard into existing workflows)	**Time (≈) → climate (≈)** The dashboard was seen as a priority; however, the staff did not have sufficient time to manage it on the same clinic day. The interviewee used downtime time from other clinic days to manage the intervention—relying on downtime (slack resources) did not have a significant influence on the implementation climate	Sustainment	“If VA’s prescribing it, we have to be responsible and do it the right way… because it’s part of our patient care, it’s supposed to be part of our clinic day, but again it’s not always feasible. So, I use the downtime that I have in my other clinic so I can go in and manage it” Site 38, Study ID 156

In the early implementation phase, one interviewee, who was a program manager, was aware of the need for more staff to keep up with the DOAC Dashboard due to a significant increase in the number of patients switching from warfarin to DOACs. Over time, the participant’s clinical site had increased the number of pharmacists working on the intervention as a result of the VA’s policy change (the Diffusion of Excellence Initiative). Despite the increase in human resources in the sustainment phase, the interviewee and colleagues still had difficulties integrating the DOAC Dashboard into their existing workflow due to the lack of a dedicated team and time.

One interviewee from another site also discussed the issue of the lack of dedicated time. The staff needed to use their downtime at another clinic to manage the dashboard. Although the staff was still able to maintain the dashboard regularly, the use of the staff’s downtime did not significantly influence the implementation climate in this case.

#### Group 3: Sites with negative changes in both resources and implementation climate

Lastly, we examined the clinical sites where available resources and implementation climate changed in a negative way over time (Table 3). One interviewee wished there was technological support to streamline the dashboard and other tools into one system to make the tool more implementable. Although the interviewee was aware of the potential benefits of using the intervention, time constraints throughout the implementation process appeared to be a major barrier to integrating the tool into workflows and caused the staff to experience guilt and burnout at work.

**Table 3:** Examples of resource-climate interactions at group 3 clinical sites

Available resources	Implementation climate	Interplay	Phase	Illustrative quote
Technological support	Compatibility (integration of the dashboard into existing workflows and systems)	**Technology (≈) → compatibility (↓)** The intervention was not able to be integrated into the daily schedule at the clinic. The interviewee suggested that they needed technological support to streamline the dashboard and other tools into one system	Implementation	“I think, we’re trying to right now work it into your daily schedule of what you’re doing would not work. Maybe if it was like part of our notes or as I said before, incorporated or integrated into our notes that we do, and at the same time I’m doing a note, I can be doing a dashboard thing, like if it was all kind of together in one, I don’t know how you’d do that, like in one program or something or if we could do a note through the dashboard that would… take everything off instead of doing it through CPRS or something maybe, but making it accessible and second nature to use, not like a separate work that you have to do on top of all the other junk that you’re doing” Site 24, Study ID 136
Time	Compatibility	**Time (↓) → compatibility (↓)** Time constraints throughout the course of implementation made it difficult to integrate the intervention into their workflows	Implementation and sustainment	“It’s not like, I don’t think it’s [dashboard] not helping, it’s not a tool, it’s just another thing to have to do and… I don’t need any more to do at this point. I’m not trying to diss, I know it’s a great idea, I sound like a slacker, but I’m really, I’m really not, I know it’s a great thing and it would be ideal if we could have it, working on it all the time but that doesn’t happen.” Site 24, Study ID 136
Consistent support from implementation partners	The extent to which the use of the intervention was supported within their organization	**Support (↓) → climate (↓)** Over time, the staff faced challenges working with physicians and felt the time they spent on the intervention was not valued and they could not make any influence on patient care through using the dashboard	Sustainment	“There are a lot of complaints about the notes I put in [sending notes too early] and there are a lot of times where the notes don’t even get signed. So, whether or not the Primary Care providers are really looking at these notes or really putting in renewals, a lot of the time they don’t. So, it’s almost like I’m wasting my time.” Site 40, Study ID 169

Another critical aspect of the interaction between available resources and implementation climate was the relationship between the support from implementation partners (physicians) and the intervention users’ motivation, which was also a theme that emerged from the interview data of group 1. One interviewee felt that the time they spent on the intervention was not valued as the physicians did not often sign their notes about medication renewals or changes. Even though the pharmacists identified potential medication errors from the dashboard, they still needed collaboration and support from physicians to make changes in patients’ medications. When the physicians chose to ignore the notes, the pharmacists and nurses could not see any difference they made to patient outcomes through the intervention use and would not feel motivated in the process.

### Summary: the dynamics of available resources across phases

Our findings of the dynamics of key resources across implementation phases are summarized in Table 4. Technological resources and champions played critical roles in facilitating users’ adoption of the technology-based intervention. Sufficient time and staffing were key resources throughout the implementation process. Having time to experiment with the new tool and to establish the ideal practice setting laid a solid foundation for the sustainment of the intervention. Our interview data showed that having more staff and time was not necessarily sufficient to guarantee success; the organizations needed time and staff specifically dedicated to intervention use in the sustainment phase. The deployment of time and staff often required support from leadership at the organizations. Consistent support and cooperation provided by implementation partners were essential to maintain users’ motivation over time.

**Table 4:** The dynamics of available resources across phases

Phase	Available resources					
	**Technology**	**Time**	**Staff**	**The support of stakeholders**		
				**Leaders**	**Champions**	**Implementation partners (e.g., physicians)**
Implementation	+ +	+ +	+ +	+ +	+ + +	+
Sustainment	+	+ + (dedicated time)	+ + (dedicated staff)	+ +	+	+ + +

## Discussion

To our knowledge, this is the first empirical study which (1) examines the dynamics of available resources and their influence on the implementation climate of an intervention across the implementation and sustainment phases and (2) contributes to a better understanding of the changing needs for resources and support over the course of implementing technology-based interventions in healthcare settings.

### The dynamic nature of available resources

Using the CFIR rating system, we assessed the levels of resource availability and the implementation climate across phases of the implementation program. We found that users’ resource needs were not static; instead, both the quantity and types of resources required for supporting the users of the intervention shifted throughout the phases of the implementation process. In the implementation phase, users required technological resources that streamlined the integration of the tool into their existing systems; in addition, they needed the support of champions to help them overcome apprehension and build trust in the new technological tool. In the sustainment phase, participants expressed the need for collaborative relationships and support from implementation partners (physicians) to implement the medication appropriateness recommendations. In some cases, the lack of support from the physicians diminished users’ motivation over time.

Most cases in this comparative study were in line with previous research that demonstrated a positive association between resource availability and implementation climate [36–38]. Nevertheless, we also identified instances where increased resources did not guarantee a more positive implementation climate over time. For example, one site with increased human resources involved in the intervention use did not result in a more positive implementation climate in the sustainment phase because the staff and time were diverted to competing priorities. Our findings echo those of previous studies that have shown the importance of dedicated resources in the sustainment of interventions [39, 40].

### Support beyond the technology

To implement the technology-based intervention, the intervention users needed different types of support that went beyond the technical aspects of an intervention and varied over time. Specifically, interview participants pointed out that social and emotional support provided by the champions was critical during the adoption and implementation of the new technological intervention. This finding strongly resonates with recent research that highlights the anxiety and burnout experienced by healthcare professionals during the adoption and utilization of EHRs and health information technology and underscores the importance of providing appropriate support to address these challenges [41, 42].

For many of the pharmacists who first adopted the DOAC Dashboard, it was a huge transition from monitoring individual patients to using a technology-based population health management approach that relies on data analytics to identify patients prescribed DOAC medications and to screen for any potential prescription red flags (e.g., inappropriate dosing for a given renal function). Since the intervention entailed a very different mechanism to identify and manage potential medication errors, pharmacists needed an in-depth understanding of the logic of the dashboard, and it took time for the pharmacists to verify the accuracy and ensure the intervention provided patients with safe and effective medication use, which is the core of their professional identity [43]. The support from champions included helping users understand the rationale behind the dashboard codes, sharing successful experiences and benefits associated with using the intervention, empathizing with users, and relating to their frustrations during the initial implementation phase.

The collaboration between the intervention users and other implementation partners was an important theme in our interview data. The implementation partners (the primary care physicians) have played a critical role in making the changes recommended by the anticoagulation professionals. As one interviewee pointed out, increasing resources, such as enhancing provider education in these clinical sites, may encourage prescribers to adopt recommendations for safer DOAC monitoring. Additionally, establishing proactive measures to promote mutual respect and organizational norms for effective communication and collaboration between pharmacists/nurses and prescribers during the medication review process may further encourage prescribers to embrace safer DOAC prescribing recommendations. By fostering and maintaining collaboration among stakeholders, the pharmacists would feel more motivated to use and sustain the intervention, ultimately driving positive changes in patient care.

### Study limitations

This study has important limitations that must be considered when interpreting the results. First, it relied on self-reported information about the implementation process and without on-site observations, which would have been impractical. We also acknowledge the unavailability of policy/practice documentation or other types of data for the purpose of triangulation. Second, applying the CFIR scoring approach to qualitative interviews may unavoidably result in the omission of certain nuanced aspects of the qualitative data. Third, we focused mainly on the implementation and sustainment phases, and especially on the front-line health professionals’ experiences with the intervention. We did not examine how available resources were planned and allocated during the pre-implementation phase, which often occurred at the higher level of the organization. Fourth, we discussed the role of leadership in the implementation from the perspectives of the participants. Additionally, the views of the intervention from patients were also beyond the scope of this study. Fifth, the findings of this study were drawn from a technology-based intervention and their applicability to a non-technology implementation intervention remains to be studied. Finally, this study was conducted in the United States VA health system, and results may not be generalizable to the settings outside of the VA or the USA.

Despite the limitations, this study highlights the dynamic nature of available resources and their impacts on the implementation climate across different phases of implementation. A more nuanced understanding of the dynamics of available resources over time from the users’ perspectives will allow adaptation to the resources to better meet the needs of the intervention stakeholders. Future studies can further explore the factors that affect physicians’ implementation of pharmacists’ recommendations and the strategies to facilitate collaboration between physicians and pharmacists.

## Conclusions

This study demonstrates the dynamic interactions between available resources and implementation climate across implementation phases. Combining the determinant and process approaches, we examined the changes in and interactions between available resources and implementation using stakeholder interviews. Our findings showed that the resources necessary to support the successful implementation of an intervention are not static; instead, both the quantity and types of resources shift based on the phases of the intervention. This study highlights the importance of different types of support for intervention users from the implementation phase to the sustainment phase. In the implementation phase, users needed technological support that facilitates the integration of the intervention into users’ existing system and social/emotional support to help them overcome apprehension and develop trust in adopting the new technological intervention. In the sustainment phase, the support from other implementation partners, especially the physicians, greatly affected whether the intervention could produce a positive outcome in patient care. Fostering and maintaining the collaboration between users and other implementation partners could help the users stay motivated during the sustainment phase.

## Supplementary Information

**Additional file 1.**

## Data Availability

The datasets generated and/or analyzed during the current study are not publicly available due to the regulatory compliance of the Department of Veterans Affairs.

## Abbreviations

CFIR: Consolidated Framework for Implementation Research
DOAC: Direct oral anticoagulant
EHR: Electronic health record
PARIHS: Promoting Action on Research Implementation in Health Services
QUERI: Quality Enhancement Research Initiative
VA: Veterans Health Administration
